# Potential Functions and Transmission Dynamics of Fungi Associated with *Anoplophora glabripennis* Across Different Life Stages, Between Sexes, and Between Habitats

**DOI:** 10.3390/insects16030273

**Published:** 2025-03-05

**Authors:** Qing Liu, Yuanting Jia, Yishuo Li, Shilong Geng, Yanqi Yu, Zhangyan Wang, Xinru Wang, Ningning Fu, Jianyong Zeng, Xiaoyu Su, Huiping Li, Hualing Wang

**Affiliations:** 1 College of Forestry, Hebei Agricultural University, Baoding 071001, China; 2 Key Laboratory of Forest Germplasm Resources and Protection of Hebei Province, Hebei Agricultural University, Baoding 071001, China; 3 Hebei Urban Forest Health Technology Innovation Center, Hebei Agricultural University, Baoding 071001, China

**Keywords:** *Anoplophora glabripennis*, different life stages, fungal community, source tracker analysis

## Abstract

The fungi residing in the gut and associated habitats play a crucial role in the growth and development of *Anoplophora glabripennis* (Motschulsky) (Coleoptera: Cerambycidae), a wood-boring pest. Yet, how they are acquired and maintained across generations, and their respective roles throughout the life cycle, remain unknown. To this end, we used high-throughput ITS sequencing analysis to characterize the fungal composition and diversity associated with *A. glabripennis* across three different life stages, between sexes, and between its habitats. Overall, the fungi composition was stage specific, with adult gut communities being more diverse than those of larvae and eggs. Male fungal communities differed significantly, while frass and female communities were more similar to each other. The top 10 most abundant genera were investigated, with *Fusarium* consistently observed in all samples and exhibiting the highest overall abundance. Function predictions revealed the presence of potentially beneficial fungi that may support *A. glabripennis* invasion across all groups. Additionally, we observed complex network structures in the fungal communities associated with eggs and males, and stronger positive correlations in those of eggs and newly hatched larvae. Source tracking analysis suggested that these fungi were vertically transmitted, following a transmission pathway of ‘female gut–frass–egg–larval gut’, occurring via frass deposited in oviposition sites. Our findings provide a nuanced understanding of the intricate interactions among plants, insects, and fungi, shedding light on the acquisition, maintenance, and roles of gut-associated fungi in *A. glabripennis*.

## Simple Summary

The Asian longhorned beetle (ALB), *Anoplophora glabripennis*, is a highly destructive wood-boring insect that poses significant economic threats. Despite the crucial role of gut-associated fungi in ALB growth and development, research on their acquisition, maintenance, and functions throughout the beetle’s lifecycle remains limited. To address this gap, we characterized the fungal composition and diversity in ALB across three distinct life stages, between sexes, and between its habitats, while exploring their potential functions. Our findings indicate that the gut fungal communities of ALB vary, with adults exhibiting greater diversity than larvae and eggs. Newly hatched larvae and eggs showed greater similarity in their gut fungal communities. Notably, *Fusarium* was consistently detected across all samples, and we identified potential beneficial fungi that may facilitate the invasion of *A. glabripennis* populations. Furthermore, we demonstrated a transmission pathway following the route of ‘female gut–frass–egg–larval gut’. These results enhance our understanding of the complex interactions between female and male beetles and their associated fungal communities.

## 1. Introduction

Beetles and fungi have a considerable degree of complexity and a diverse relationship [1,2]. The fungi are present throughout the various life stages of beetles, co-evolving with their hosts to form intricate and specific interactions that serve a variety of functions [1]. It has been widely documented that symbioses can promote beetle growth [3], supply nutrition [4,5,6,7], create resistance to pathogen colonization [8,9,10,11,12], and produce signaling molecules [13,14,15]. In return, insects offer the fungi several advantages, including a long-term stable habitat and the utilization of shared metabolic pathways [16]. The evolutionarily mutualistic symbiotic relationship between them plays a crucial role in the organization and functioning of communities [17,18]. For several beetle species, the maintenance of symbionts through vertical transmission is crucial for their long-term survival and reproductive success [19,20,21,22]. Maternal microbiota transfer occurs via several different mechanisms, including through capsules, intracellularly, and through secretions and excretions [23,24,25,26]. Besides maternal transfer, insects can also acquire environmental microbiota from food or soil [27,28], which enhances their performance and fitness. It is plausible that maternal acquisition and environmental acquisition are intersecting processes, especially when considering symbionts that inhabit both the insect gut and external environments [29].

The Asian longhorned beetle (ALB, *Anoplophora glabripennis*) is a wood-feeding insect that attacks and kills a variety of tree species [30,31], including maple (*Acer* spp.), poplar (*Populus* spp.), and willow (*Salix* spp.) [30,31,32]. Adults feed on the bark, leaves, and petioles until they reach reproductive maturity, at which point they seek to complete mating [13,14]. During oviposition, the female adult chews through the bark to reach the phloem (hereafter referred to as an oviposition slit) and lays eggs into the phloem–cambium interface, then deposits frass into the oviposition slit [29,33]. Newly hatched larvae feed on the phloem and subsequently bore into the heartwood, disrupting the vascular tissue [34]. Although the nutritional content of the phloem, xylem, and heartwood may differ, they are all considered nutritionally poor substrates [35,36]. The role of the microbiota from the gut and the associated habitats in providing nutrition to the *A. glabripennis* is of particular significance [37,38,39,40,41].

Recently, numerous studies have highlighted a correlation between the structure and composition of the gut fungi in insects and their various developmental stages. Specifically, research conducted on *Agrilus mali* (Matsumara) (Coleoptera: Buprestidae) has demonstrated significant changes in the gut microbiota as the insects progress from the larval and pupal to the adult stage [42]. Similarly, in *Trypophloeus klimeschi* (Eggers) (Coleoptera: Curculionidae), the fungi genera *Nakazawaea*, *Trichothecium*, and *Aspergillus* emerge as predominant throughout the larval gut, whereas *Graphium* dominates in the adult stage [43]. Moreover, the gut fungi at each developmental stage plays a crucial role in facilitating the host’s adaptation to and management of environmental changes. For instance, in *Bactrocera dorsalis* (Hendel) (Diptera: Tephritidae), *Hanseniaspora uvarum* (Niehaus) (Ascomycota: Saccharomyceta) has been shown to shorten the larval development time and increase the adult wing length, as well as to enhance the pupal and adult body size and weight [44]. Although our understanding of gut fungi in insects has advanced [6,8,15,42,45,46,47,48], there remains a significant gap in our knowledge regarding the population dynamics and potential functions of fungal communities across the different life stages of *A. glabripennis* and its associated habitats, despite some exploratory studies. This research area is crucial, as it can provide insights into the stability and potential vertical transfer of microbial communities from mothers to offspring. Mason et al. (2019) [29] observed that, despite variation in the presence and abundance of individuals from different samples, *A. glabripennis* was consistently associated with the *Fusarium solani* (Hypocreales: Nectriaceae) species complex (FSSC) in *Acer rubrum* L. (Sapindales: Aceraceae). They also found that frass can act as a vector for the dissemination of the gut microbiota. While diet is known to have a profound effect on the composition of fungal communities in the gut [39,42,49], the variations observed in *A. glabripennis* on *Salix matsudana* (Koidz) (Salicales: Salicaceae) remain unclear, and it is uncertain whether similar trends exist in other contexts.

In this study, we employed high-throughput ITS sequencing, a co-occurrence network, and source tracking analysis to conduct a comprehensive fungi community analysis at different life stages and in associated habitat samples. Our objectives were threefold: (1) to evaluate and compare the fungal community composition among the various life stages of *A. glabripennis* and the fungi present in its habitat; (2) to identify the dominant fungi and their potential functions in different samples; and (3) to determine the sources of the gut fungi carried by the larvae.

## 2. Materials and Methods

### 2.1. Collection and Processing of Samples

In June 2023, the adults of *A. glabripennis* and the oviposition slit samples were collected in Xiong’an, Hebei Province, China (116°4′15.14″ E, 39°5′6.61″ N), and then were transported back to the laboratory. The adults were placed in insect rearing cages at a 1:1 male-to-female ratio, with fresh *S. matsudana* branches provided daily at a temperature of 25 ± 1 °C and a relative humidity of 65 ± 10% [50]. Fresh frass was collected from the female insects daily. Similarly, fresh eggs were also collected daily from the branches. A subset of eggs were stored in sterile tubes, while the remaining eggs were placed on moistened filter paper and incubated in a location shielded from direct sunlight to collect newly hatched larvae. The larvae were then rinsed repeatedly with sterile water, immersed in a 75% alcohol solution for two minutes, rinsed again with sterile water, and immediately dissected to extract their guts. Thus, a total of 29 samples were selected for the study: 4 female guts, 3 male guts, 6 oviposition slits, 6 frasses, 4 eggs, and 6 larval guts. All samples were rapidly frozen in liquid nitrogen and stored at −80 °C until they were processed for DNA extraction.

### 2.2. DNA Extraction and Sequencing Analysis

Total DNA was extracted from the tissue samples from eggs, and the female gut, male gut, larval gut, frass, and oviposition slit using the E.Z.N.A.^®^ Stool DNA Kit (Omega Bio-Tek, Norcross, GA, USA). For fungal ITS amplification, all DNA samples were adjusted to the same concentration and the primers ITS1F (5′-CTTGGTCATTTAGAGGAAGTAA-3′) and ITS2R (5′-GCTGCGTTCTTCATCGATGC-3′) were employed [51]. For each sample, a 20 µL PCR reaction mixture was prepared, containing 4 µL of 5× FastPfu Buffer, 2 µL of 2.5 mM dNTPs, 0.8 µL each of forward and reverse primer, 0.4 µL of FastPfu Polymerase, 10 ng of template DNA, and ddH_2_O to reach the final volume. The PCR amplification conditions were as follows: 95 °C for 5 min, followed by 30 cycles at 95 °C for 30 s; 55 °C for 30 s; 72 °C for 45 s; and 72 °C for 10 min. The PCR products were then purified using the AxyPrep DNA Gel Extraction Kit (Axygen Biosciences, Union City, CA, USA) and quantified using Qubit^®^3.0 (Life Technologies, Carlsbad, CA, USA). Positive and negative controls were incorporated to ensure the accuracy and reliability of our results. The genomic DNA library was constructed using the Illumina Paired-End library construction method. Subsequently, the amplicon libraries were sequenced using paired-end (2 × 250) technology on the Illumina Novaseq 6000 platform [52], which was provided by Shanghai BIOZERON Co., Ltd. (Shanghai, China), following established protocols. Sequencing analysis adhered to the methodologies outlined by Mogouong et al. (2021) [53] and Koski et al. (2024) [54].

### 2.3. Sequencing Data Analysis

The passed sequences underwent dereplication and were processed using the DADA2 algorithm (recommended by QIIME 2) to detect indel mutations and substitutions [55]. Paired reads were trimmed and filtered, allowing for a maximum of two expected errors per read (maxEE = 2). Following the merging of the paired reads and chimera filtering, the phylogenetic affiliation of each ITS gene sequence (hereinafter referred to as ASVs) was determined using the RDP Classifier (https://github.com/rdpstaff/classifier, accessed on 2 October 2023.) against the UNITE database, with a confidence threshold set at 70% [56]. Alpha diversity analyses of fungi were performed separately from different life stages and habitats using the Mothur software (v.1.30.1) [57], including the Chao1, ACE, and Shannon and Simpson indices. To assess the differences in the fungal community structure of the samples, all samples were subjected to beta diversity using Principal Co-ordinates Analysis (PCoA) based on the Bray–Curtis distance at the genus level. To detect significant differences in fungi diversity, one-way ANOVA was performed using SPSS software (v.29.0).

Functional prediction analyses of the fungal communities were performed using the FunGuild database [58]. Heatmaps were subsequently generated to illustrate the top 20 subfunctions, ranked by their relative abundance. To further explore the interactions among the fungal genera, Pearson correlation analysis was then performed on the top 50 most abundant fungal genera. The analysis revealed correlation coefficients exceeding 0.6 with *p*-values below 0.01, indicating strong and significant correlations. Based on these findings, network construction was carried out using Gephi software (v.0.10.1) [59] to visualize the interactions. Additionally, we used SourceTracker (v1.0), available at https://github.com/danknights/sourcetracker (accessed on 30 October 2024.), to investigate the origins of the fungi found in the guts of newly hatched larvae [60].

## 3. Results

### 3.1. Diversity of Fungal Communities

A total of 29 samples were sequenced, yielding 1,307,639 clean reads after the quality control. The sequencing coverage exceeded 99%, indicating that the sequencing data accurately represented the diverse fungal communities present in the samples (Table S1).

**Figure 1:**
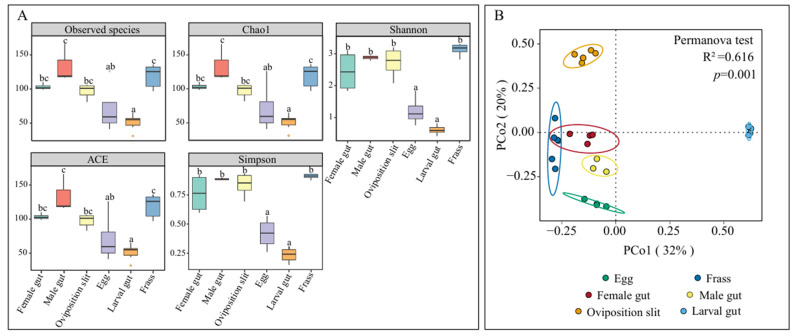
Alpha diversity (**A**) and beta diversity (**B**) indices of fungal communities in different life stages and habitats of *A. glabripennis*. a–c indicated significant difference among samples at the *p* < 0.05 level.

The richness and diversity of the fungal ASVs varied across the *A. glabripennis* life stages and associated habitats. Differences in alpha diversity indices among the six groups were analyzed using ANOVA (Figure 1A). The highest fungal diversity was observed in the males and frass, followed by the female and oviposition slit. A clear trend of gradually decreasing α diversity was evident from the eggs and newly hatched larval guts. PCoA analysis revealed distinct fungal compositions among the six groups. Samples from the oviposition slit, male guts, newly hatched larval guts, and eggs formed distinct and significantly different clusters. Additionally, the frass and female groups exhibited partial overlap (R^2^ = 0.616, *p* = 0.001), indicating that their fungal communities were more similar to each other than to the other samples (Figure 1B).

### 3.2. Dominant Fungal Community Composition

**Figure 2:**
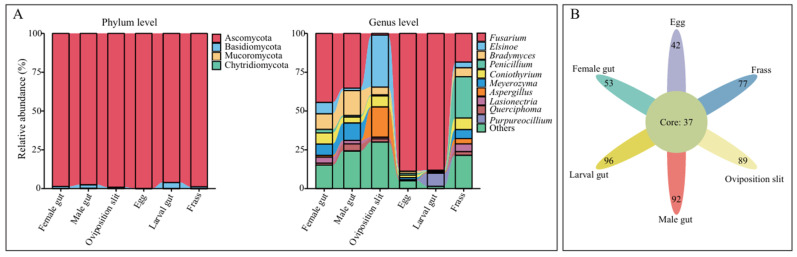
Community structure of fungi in the different life stages and habitats of *A. glabripennis* at the phylum and genus levels (**A**). Core gut microbiota of *A. glabripennis* (**B**). Venn diagram illustrating the number of common fungi shared among different groups.

Across all samples, the 897 Amplicon Sequence Variants (ASVs) were assigned to 4 fungal phyla, 22 classes, 55 orders, 113 families, 225 genera, and 413 species. Of them, Ascomycota was the most abundant phylum, with a relative abundance exceeding 95% in all groups (Figure 2A). At the genus level, we observed that *Fusarium* was the most abundant genus in both females (44.43%) and males (33.27%), followed by *Bradymyces* (10.04% in females, 16.05% in males) and *Meyerozyma* (7.29% in females, 11.29% in males) (Table S2). In the oviposition slit, the most prevalent genera were *Elsinoe* (33.55%), *Aspergillus* (19.45%), and *Coniothyrium* (7.06%). A notable shift was observed in the eggs and newly hatched larval gut, where *Fusarium* dominated, accounting for more than 80% of the relative abundance. In the frass, the top three most abundant genera were *Penicillium* (26.59%), *Fusarium* (18.38%), and *Coniothyrium* (7.42%). In terms of ASV richness, the newly hatched larval gut had the highest number of fungal ASVs, followed by males, the oviposition slit, frass, females, and eggs. A total of 37 common ASVs were identified across the six groups (Figure 2B). The overall differences in fungal composition among the stages and habitats were evident. However, the newly hatched larval gut and eggs exhibited a greater similarity compared to the adults. Notably, *Fusarium* was present in all samples.

### 3.3. Differential Fungal Communities

**Figure 3:**
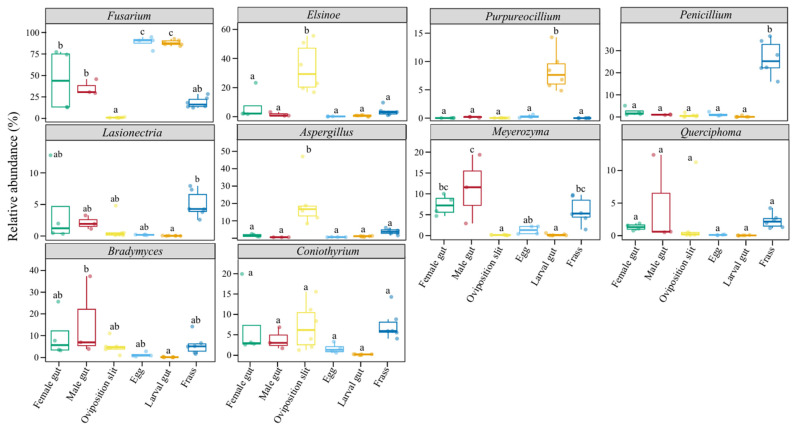
Distinct genera of fungi in the different life stages and associated habitats of *A. glabripennis*. Different letters indicate significant differences among samples at the *p* < 0.05 level, respectively.

To gain further insight into the variations in fungi across different life stages and associated habitats, we examined the dynamics of the top 10 most abundant genera (Figure 3). It is noteworthy that *Fusarium* was present in all six sample groups, with its highest abundance observed in the egg and newly hatched larval gut groups. In contrast, its abundance was only half as high in the female and male groups. Remarkably, among the four groups, *Fusarium* exhibited the highest overall abundance. *Aspergillus* and *Elsinoe* were the predominant fungal genera in the oviposition slit group, while *Penicillium* was the most abundant in the frass group. Furthermore, *Purpureocillium* was significantly more prevalent in the newly hatched larval gut group compared to the other samples.

### 3.4. Potential Functions of the Microbiome in Fungal Communities

**Figure 4:**
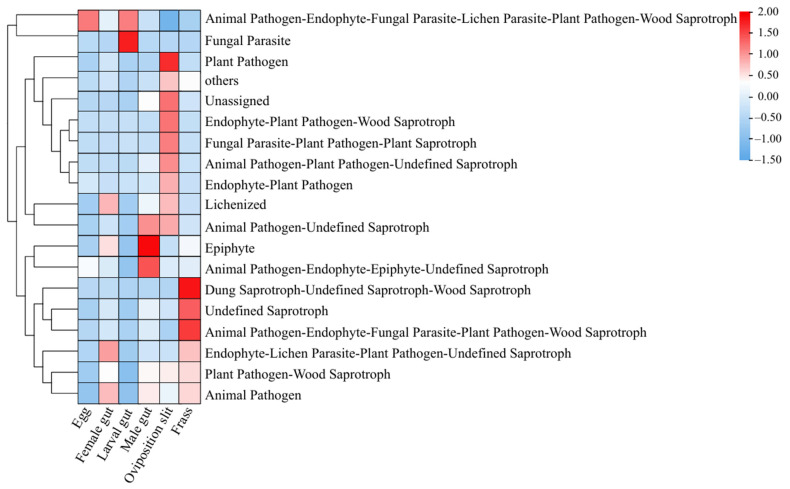
Functional prediction of fungi across different life stages and the associated habitats of *A. glabripennis*.

Our results reveal distinct fungal functionalities across all six groups (Figure 4). In the eggs group, the predominant fungal function was Animal Pathogen–Endophyte–Fungal Parasite–Lichen Parasite–Plant Pathogen–Wood Saprotroph (7.11%), primarily associated with the genus *Fusarium* (42 ASVs). For the newly hatched larval gut group, the primary function was Fungal Parasite (0.79%), corresponding to the genus *Purpureocillium* (six ASVs). In the male gut groups, the dominant function was Epiphyte (6.05%), linked to the genus *Bradymyces* (36 ASVs). For the female gut group, the predominant function was Endophyte–Lichen Parasite–Plant Pathogen–Undefined Saprotroph (1.97%), corresponding to the genus *Alternaria* (15 ASVs). The oviposition slit group exhibited the most prominent function of Plant Pathogen (6.97%), associated with the genus *Acremonium* (8 ASVs), and was associated with Animal Pathogen–Endophyte–Fungal Parasite–Plant Pathogen–Wood Saprotroph (1.05%). In the frass group, the primary function was Dung Saprotroph–Undefined Saprotroph–Wood Saprotroph (3.68%), linked to the genus *Penicillium* (28 ASVs). Overall, the dominant fungi in the various life stages and habitats of ALB are largely associated with plant and animal pathogens, saprophytes, and wood saprotrophs.

### 3.5. Fungal Interactions Revealed by Co-Occurrence Network

**Figure 5:**
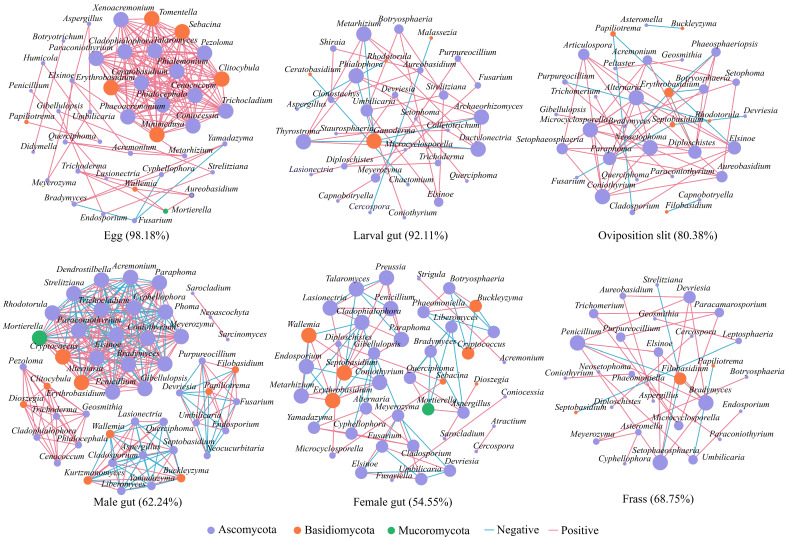
Correlation network analysis of fungi in the different life stages and associated habitats of *A. glabripennis* at the genus level. Different colors denote distinct phyla. The sizes of the shapes indicate the degree of connectivity, and the lines connecting the points signify the relevance between them. Specifically, the red lines represent a positive correlation, whereas the blue lines indicate a negative correlation, and percentage indicates positive correlation ratio.

We conducted a comprehensive co-occurrence network analysis to explore fungal interactions. The network was dominated by Ascomycota, accounting for over 79% of all samples (Figure 5). Notably, the networks of eggs (42 nodes, 168 edges) and males (49 nodes, 294 edges) exhibited more complex structures compared to the other four groups. In both the eggs and newly hatched larval gut groups, we observed stronger positive correlations among fungi (98.18% and 92.11%, respectively). The female group comprised 43 nodes and 77 edges, exhibiting a relatively low positive correlation rate of 54.55% and included five core genera. The oviposition slit group had 33 nodes and 51 edges, with a positive correlation of 80.38%, and included four core genera: *Alternaria*, *Coniothyrium*, *Microcyclosporella*, and *Neosetophoma*. The frass group demonstrated an overall low positive correlation of 68.75%, with 29 nodes and 32 edges, and comprised three core genera: *Bradymyces*, *Penicillium*, and *Setophaeosphaeria* (Table S3). Overall, the complex network structure observed in the eggs and males suggests a high degree of microecological stability, while the stronger positive correlations in the eggs and newly hatched larval gut indicate predominantly collaborative interactions among fungi.

### 3.6. The Potential Transmission Processes of ALB Gut Fungi

**Figure 6:**
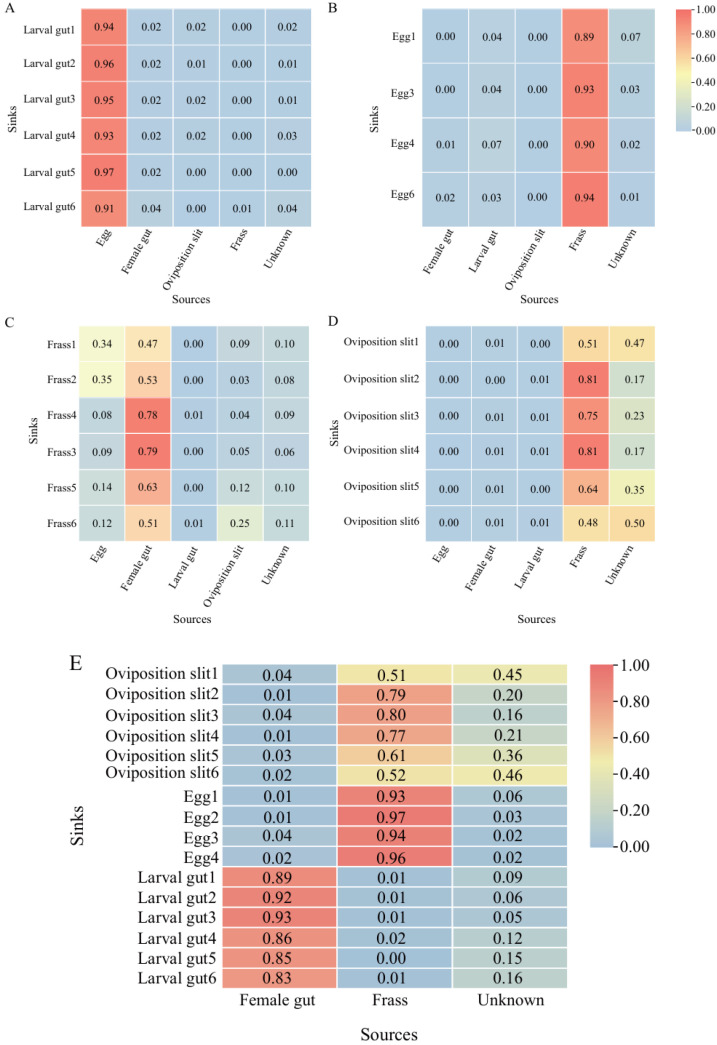
Source tracing measurements of microbial fungi in the newly hatched larval gut. (**A**) Predicted proportions of larval gut microbiota originating from eggs, female gut, oviposition slit, and frass. (**B**) The proportion of microbiota in eggs originating from the female gut, larval gut, oviposition slit, and frass was estimated. (**C**) Proportions of microbiota in frass samples originating from eggs, female gut, larval gut, and oviposition slit. (**D**) Microbiota in oviposition slit samples derived from eggs, female gut, larval gut, and frass. (**E**) Proportions of microbiota from female gut and frass samples transferred to larval gut, frass, and oviposition slit. The numbers represent the compositional proportions of the samples from each source in the sink samples. The larger the proportion, the redder the color; conversely, the smaller the proportion, the bluer the color.

**Figure 7:**
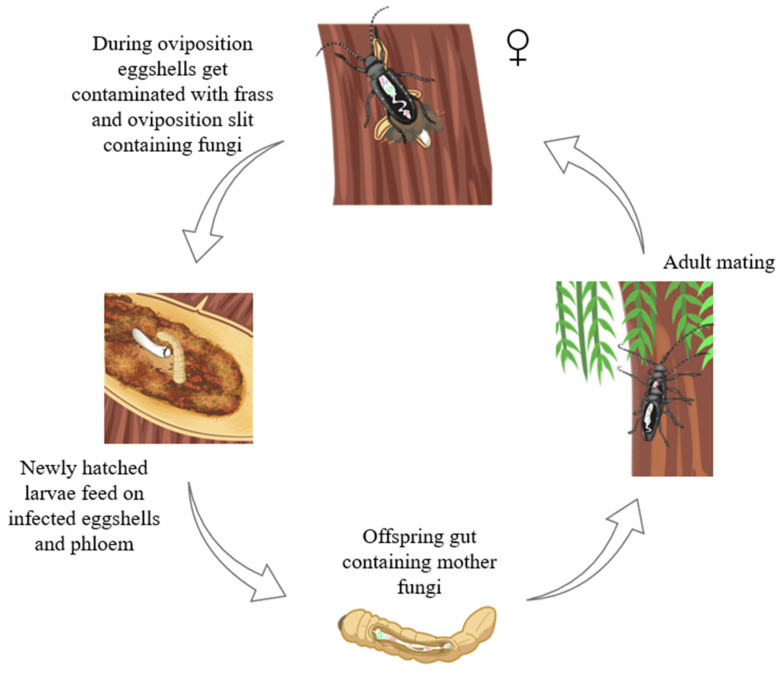
Schematic illustrating the transmission of the fungi from mother to offspring, which occurs via frass deposited during oviposition.

The source tracking result showed that when the newly hatched larval gut was designated as the sink, 94.33% of the fungi was sourced from eggs (female gut, 2.08%; oviposition slit, 1.28%; frass, 0.41%) (Figure 6A). Frass sources contributed 91.50% of the egg fungi (female gut, 0.95%; newly hatched larval gut, 0.45%; oviposition slit, 0.16%) (Figure 6B). When examining the frass as the sink, 66.67% of the fungi in the frass (egg, 18.78%; larval gut, 0.32%; oviposition slit, 9.90%) came from female gut (Figure 6C). Likewise, 66.67% of oviposition slit fungi originated from frass sources (egg, 0.17%; female gut, 6.06%; larval gut, 0.50%) (Figure 6D). The assignment of the female gut and frass as sinks, simultaneously, revealed that the female gut contributed 88% to the larval gut fungi, while frass contributed 95% to the egg fungi. Additionally, 66.67% of the oviposition slit fungi were identified as originating from frass (Figure 6E). Overall, the above results suggested that frass can serve as a vehicle for the transmission of a subset of the maternal gut fungi. Therefore, we present a schematic in Figure 7 that illustrates the spread of the ALB fungus, demonstrating that the fungi transmission pathway was ‘female gut–frass–egg–larval gut’.

## 4. Discussion

Fungi play a vital role in the growth and development of insects, as well as in the health of plants [61]. Notably, the fungal community composition of the ALB varies across its different life stages and the associated habitats, with not all adult fungal taxa being present in the larval gut (Figure 2). This suggests that some components of the adult fungi may be unstable, unable to colonize the larval gut or wood, or are eliminated during the colonization process [29]. This ultimately results in the relatively low alpha diversity of fungi in the egg and larval gut (Figure 1A), a characteristic that is also commonly observed in other Coleoptera species [62,63,64].

The various life stages and habitats of *A. glabripennis* were predominantly associated with Ascomycota (Figure 2), which is also recognized as the most prevalent phylum in the majority of Coleoptera individuals [65]. When *S. matsudana* served as the host, we found *Fusarium* was present in the various stages and the associated habitats of *A. glabripennis*, suggesting it acts as a fungal symbiont (Figure 2 and Figure 3). In line with our results, *Fusarium* has been reported as a well-known symbiotic fungi of Coleoptera found in leaf beetles, ambrosia-boring beetles, and wood-boring beetles [66,67,68]. Additionally, members of the *Fusarium*, with *F. solani* being the most extensively studied species within the members of FSSC [69,70], are significant pathogens of numerous agriculturally important crops [71], linked to a range of plant diseases, including wilt and rot [72,73,74,75,76,77,78]. In this study, it was found that the larval gut harbored the highest abundance of *Fusarium*, presumably because the fungi assist the larvae in acquiring the essential nutrients required for their growth and development [79,80]. Consistent with this explanation, *F. solani*, isolated from the gut of ALB larvae, consistently produces a reliable lignocellulose enzyme and nourishes larvae, leading to increased weight and head capsule width. This highlights its pivotal role in the survival and growth of subsequent generations [81].

The dominant fungal functions at various life stages and habitats of ALB are primarily linked to plant and animal pathogens, as well as saprotrophs (Figure 4). A significantly higher concentration of *Elsinoe* was observed in the oviposition slit (Figure 3). This genus comprises a wide range of aggressive plant pathogens that can induce scab and spot anthracnose on numerous plant species [82,83], including economically important ones, such as avocado [84], citrus [85], vines [86], and woody plants [87,88]. Zhao et al. (2020) [87] have confirmed *Elsinoe australis* (Myriangiales: Elsinoaceae), isolated from *Populus tomentosa* (Carrière) (Malpighiales: Salicaceae) and *Populus deltoides* (Marshall) (Malpighiales: Salicaceae) leaves, to be a pathogen through in vitro pathogenicity tests. However, the specific role of the high abundance of this genus in the oviposition slit remains to be elucidated.

*Aspergillus* and *Penicillium* exhibit the highest concentrations in the oviposition slit and frass, respectively, with relatively low levels detected in the eggs and larval guts (Figure 3), as reported by Johanna Schott et al. (2024) [89]. *Aspergillus*, a saprotrophic and pathogenic fungus [90], is notably rare in the eggs, neonate larvae, pupae, and newly eclosed adults of *Chelymorpha alternans* Boh. (Coleoptera: Chrysomelidae). *Aspergillus austwickii* (Eurotiales: Aspergillaceae) stands out for its high levels of pectinase activity [91] and remarkable production of secondary metabolites, which have insecticidal properties that target insect metabolic systems [91,92]. On the other hand, *Penicillium* has been found growing on frass, where it can obtain nutrients [89]. This fungus has the ability to break down plant cell walls; for instance, *Penicillium crustosum* (Eurotiales: Aspergillaceae), found in the gut of *Saperda vestita* (Say) (Coleoptera: Cerambycidae), can degrade cellulose [93]. Furthermore, *Penicillium* can also provide its host with nutrients such as amino acids, vitamins, and sterols [17,94], and it enhances insect performance by evading or suppressing the host plant’s herbivore defenses [95,96,97,98]. Interestingly, we discovered *Purpureocillium* in larval guts (Figure 3), particularly intriguing due to its biological control and biotechnology applications [99,100,101]. This fungus not only promotes plant growth, but it also acts as a potential bioinsecticide [102,103]. Its pesticidal activity has been documented against a diverse array of pests, including those that are notoriously difficult to control with conventional chemical insecticides [104,105,106]. The presence of *Purpureocillium* in larval guts hints at a symbiotic relationship that may aid in digestion and protect against pathogens, enhancing insect survival. Future research should explore its mechanisms and potential as a sustainable insecticide alternative, with promising applications in agriculture, forestry, and public health.

After mating, the males were absent from the entire egg-laying process [3,29,64,81,107], meaning that the eggs, newly hatched larvae, and frass were all in direct or indirect contact exclusively with the females. Consequently, the fungal community structure in males differed significantly (Figure 1B). When we investigated the fungal source of the larval gut, treating the female gut, egg, frass, and oviposition slit as sinks separately, we found that the transmission pathway was ‘female gut–frass–egg–larval gut’. Furthermore, by designating the female gut and frass as sources, the female gut fungi could be transferred to the larval gut, and fungi from the frass could be transferred to the eggs (Figure 6). This further suggests that the fungi in the larval gut originate from the females and require frass for transmission [29,64]. Although there are various pathways for fungi to transfer to the eggs [23,24,25,26], and the females use the ovipositor to place the eggs under the bark, the opportunities for eggs to come into contact with other parts of the body are minimal. Therefore, the likelihood of transmission via frass is the highest.

During the transmission process, not all maternal fungi are passed on to the offspring, leading to a loss of fungal diversity (Figure 2A). Only a small subset of essential fungi is retained in the progeny [29]. As a consequence, the fungi species richness in eggs and newly hatched larvae is limited, and their ecological niches within their respective environments remain only partially colonized by fungi, all of which are in an expansionary phase. This results in fungal communities that predominantly exhibit synergistic interactions, contributing to a stable microecological environment (Figure 5, egg and larval gut). Furthermore, when compared to larval guts, eggs are immobile and have less exposure to the external environment than organisms in the oviposition slit. Consequently, eggs exhibit not only the highest positive correlation but also possess a more intricate network structure, characterized by a higher number of nodes and an increased density. Adults require supplementary nutrition to fulfill their reproductive demands [40], rendering them more susceptible to interference from exogenous environmental fungi. This susceptibility explains the relatively poor stability of their intestinal fungal microecology (Figure 5, female and male). However, the collinear network structure of male guts is more stable than that of female guts, potentially owing to the fact that females require supplementary nutrients in greater quantities for oviposition and consume more food than males, thereby being more profoundly influenced by environmental factors.

The limitations of this article stem from the fact that our investigation focused solely on the differential changes in gut fungal communities across the life stages of females, males, eggs, and the larvae of the beetle. Consequently, the changes in fungal communities during the pupal to adult eclosion phase remain an uncharted territory. In contrast, the symbiotic partnership between *Fusarium oxysporum* (Hypocreales: Nectriaceae) and the beetle *C. alternans* revealed that the fungus protects the beetle’s pupal stage against predation in exchange for being spread to its host [108]. This finding prompts an intriguing question: do the fungi associated with ALB in the pupae stage also exhibit similar protective mechanisms? Furthermore, *Fusarium* fungi commonly produce toxic secondary metabolites [109,110], which are known for their insecticidal properties. Therefore, a valuable and worthwhile direction for future research is to investigate the high tolerance of ALB to *Fusarium* and its mycotoxins, and to explore the underlying mechanisms of this tolerance [111,112,113,114,115].

## 5. Conclusions

In conclusion, our findings illustrate that the fungal community composition of the ALB varies across the lifecycle, between sexes, and between the associated habitats. Specific fungi are closely associated with *A. glabripennis*, being transmitted across generations, and the refined fungal community which is retained may be crucial for the survival and growth of the progeny. Functional predictions of microbial communities in the *A. glabripennis* lifecycle and their associated habitats on host trees suggest roles in nutrient acquisition and invasion, likely contributing to *A. glabripennis*’s ability to thrive on a wide range of host tree species. Furthermore, our study offers a comprehensive understanding of the potential sources of the larval gut fungi, highlighting that maternal microorganisms can be transmitted via frass deposited in the oviposition slit near the eggs (Figure 7). Moving forward, we plan to use culture techniques to isolate and characterize individual fungus species, which will pave the way for further exploration of the intricate relationships between microbes and insect–plant associations, which is vital for developing effective pest control strategies.

## Data Availability

The raw sequence data were deposited in the National Center for Biotechnology Information (NCBI) Sequence-Read Archive (SRA) database under accession number PRJNA1204139.

## Supplementary Materials

The following supporting information can be downloaded at: https://www.mdpi.com/article/10.3390/insects16030273/s1, Table S1: Sample-specific species ASV list and annotation summary table; Table S2: The list of identified 228 fungal genera from the nine samples; Table S3: Network topology of fungi in the different life stages and associated habitats of *Anoplophora glabripennis*.

## References

[1] Douglas A.E. (2015). Multiorganismal insects: Diversity and function of resident microorganisms. Annu. Rev. Entomol..

[2] Engel P., Moran N.A. (2013). The gut microbiota of insects-diversity in structure and function. FEMS Microbiol. Rev..

[3] Wang G., Wang X., Yang Z., Wang S., Li W., Shang S., Luo Y., Wang L. (2023). Effects of *Fusarium solani* on the growth and development of *Anoplophora glabripennis* larvae. Microb. Ecol..

[4] Ayres M.P., Wilkens R.T., Ruel J.J., Lombardero M.J., Vallery E. (2000). Nitrogen budgets of phloem-feeding bark beetles with and without symbiotic fungi. Ecology.

[5] Thompson B.M., Grebenok R.J., Behmer S.T., Gruner D.S. (2013). Microbial symbionts shape the sterol profile of the xylem-feeding wood wasp, *Sirex noctilio*. J. Chem. Ecol..

[6] Morales J.J., Zúñiga G., Villa T.L., Hernández R.C. (2009). Bacterial community and nitrogen fixation in the red turpentine beetle, *Dendroctonus valens* LeConte (*Coleoptera:* Curculionidae: Scolytinae). Microb. Ecol..

[7] Morales J.J., Vera P.L.A., García D.A., Vera P.L.A., García-Domínguez A., Martínez R.E., Zúñiga G., Hernández R.C. (2013). Nitrogen-fixing and uricolytic bacteria associated with the gut of *Dendroctonus rhizophagus* and *Dendroctonus valens* (Curculionidae: Scolytinae). Microb. Ecol..

[8] Cardoza Y.J., Klepzig K.D., Raffa K.F. (2006). Bacteria in oral secretions of an endophytic insect inhibit antagonistic fungi. Ecol. Entomol..

[9] Scott J.J., Oh D.C., Yuceer M.C., Klepzig K.D., Clardy J., Currie C.R. (2008). Bacterial protection of beetle-fungus mutualism. Science.

[10] Adams A.S., Currie C.R., Cardoza Y., Klepzig K.D., Raffa K.F. (2009). Effects of symbiotic bacteria and tree chemistry on the growth and reproduction of bark beetle fungal symbionts. Can. J. For. Res..

[11] Adams A.S., Jordan M.S., Adams S.M., Suen G., Goodwin L.A., Davenport K.W., Currie C.R., Raffa K.F. (2011). Cellulose-degrading bacteria associated with the invasive wood wasp *Sirex noctilio*. ISME J..

[12] Therrien J., Mason C.J., Cale J.A., Adams A., Aukema B.H., Currie C.R., Raffa K.F., Erbilgin N. (2015). Bacteria influence mountain pine beetle brood development through interactions with symbiotic and antagonistic fungi: Implications for climate-driven host range expansion. Oecologia.

[13] Boone C.K., Keefover-Ring K., Mapes A.C., Adams A.S., Bohlmann J., Raffa K.F. (2013). Bacteria associated with a tree-killing insect reduce concentrations of plant defense compounds. J. Chem. Ecol..

[14] Cheng C., Xu L., Xu D., Lou Q., Lu M., Sun J. (2016). Does cryptic microbiota mitigate pine resistance to an invasive beetle-fungus complex? Implications for invasion potential. Sci. Rep..

[15] Xu L., Lou Q., Cheng C., Lu M., Sun J. (2015). Gut-Associated bacteria of *Dendroctonus valens* and their Involvement in verbenone production. Microb. Ecol..

[16] Van Der Heijden M.G., De Bruin S., Luckerhoff L., Van Logtestijn R.S., Schlaeppi K. (2016). A widespread plant-fungal-bacterial symbiosis promotes plant biodiversity, plant nutrition and seedling recruitment. ISME J..

[17] Six D.L. (2012). Ecological and Evolutionary determinants of bark beetle -fungus symbioses. Insects.

[18] De Fine Licht H.H., Biedermann P.H. (2012). Patterns of functional enzyme activity in fungus farming ambrosia beetles. Front. Zool..

[19] Pons I., González Porras M.Á., Breitenbach N., Berger J., Hipp K., Salem H. (2022). For the road: Calibrated maternal investment in light of extracellular symbiont transmission. Proc. R. Soc. B.

[20] Ganesan R., Janke R.S., Kaltenpoth M., Flórez L.V. (2023). Colonization dynamics of a defensive insect *ectosymbiont*. Biol. Lett..

[21] Tetreau G., Dhinaut J., Galinier R., Audant-Lacour P., Voisin S.N., Arafah K., Chogne M., Hilliou F., Bordes A., Sabarly C. (2020). Deciphering the molecular mechanisms of mother-to-egg immune protection in the mealworm beetle *Tenebrio molitor*. PLoS Pathog..

[22] Kyei-Poku G., Gauthier D., Schwarz R., van Frankenhuyzen K. (2011). Morphology, molecular characteristics and prevalence of a *Cystosporogenes* species (Microsporidia) isolated from *Agrilus anxius* (Coleoptera: Buprestidae). J. Invertebr. Pathol..

[23] Salem H., Bauer E., Kirsch R., Berasategui A., Cripps M., Weiss B., Koga R., Fukumori K., Vogel H., Fukatsu T. (2017). Drastic genome reduction in an herbivore’s pectinolytic symbiont. Cell.

[24] Fukatsu T., Hosokawa T. (2002). Capsule-transmitted gut symbiotic bacterium of the Japanese common plataspid stinkbug, *Megacopta punctatissima*. Appl. Environ. Microbiol..

[25] Hosokawa T., Hironaka M., Mukai H., Inadomi K., Suzuki N., Fukatsu T. (2012). Mothers never miss the moment: A fine-tuned mechanism for vertical symbiont transmission in a subsocial insect. Anim. Behav..

[26] Salem H., Kreutzer E., Sudakaran S., Kaltenpoth M. (2012). Actinobacteria as essential symbionts in firebugs and cotton stainers (Hemiptera, Pyrrhocoridae). Environ. Microbiol..

[27] Kikuchi Y., Hosokawa T., Fukatsu T. (2007). Insect-microbe mutualism without vertical transmission: A stinkbug acquires a beneficial gut symbiont from the environment every generation. Appl. Environ. Microbiol..

[28] Mason C.J., Raffa K.F. (2014). Acquisition and structuring of midgut bacterial communities in gypsy moth (Lepidoptera: Erebidae) larvae. Environ. Entomol..

[29] Mason C.J., Campbell A.M., Scully E.D., Hoover K. (2019). Bacterial and fungal midgut community dynamics and transfer between mother and brood in the Asian longhorned beetle (*Anoplophora glabripennis*), an invasive xylophage. Microb. Ecol..

[30] Haack R.A., Hérard F., Sun J., Turgeon J.J. (2010). Managing invasive populations of Asian longhorned beetle and citrus longhorned beetle: A worldwide perspective. Annu. Rev. Entomol..

[31] Meng P.S., Hoover K., Keena M.A. (2015). Asian longhorned beetle (Coleoptera: *Cerambycidae*), an introduced pest of maple and other hardwood trees in North America and Europe. J. Integr. Pest. Manag..

[32] Hu J., Angeli S., Schuetz S., Luo Y., Hajek A.E. (2009). Ecology and management of exotic and endemic Asian longhorned beetle *Anoplophora glabripennis*. Agric. For. Entomol..

[33] Keena M.A., Sánchez V. (2018). Reproductive behaviors of *Anoplophora glabripennis* (Coleoptera: *Cerambycidae*) in the Laboratory. J. Econ. Entomol..

[34] Dhandapani R.K., Duan J.J., Palli S.R. (2020). Orally delivered dsRNA induces knockdown of target genes and mortality in the Asian long-horned beetle, *Anoplophora glabripennis*. Arch. Insect Biochem. Physiol..

[35] Liu F., Wickham J.D., Cao Q., Lu M., Sun J. (2020). An invasive beetle-fungus complex is maintained by fungal nutritional-compensation mediated by bacterial volatiles. ISME J..

[36] Lehenberger M., Foh N., Göttlein A., Six D., Biedermann P.H.W. (2021). Nutrient-poor breeding substrates of ambrosia beetles are enriched with biologically important elements. Front. Microbiol..

[37] Geib S.M., Jimenez-Gasco M.d.M., Carlson J.E., Tien M., Hoover K. (2009). Effect of host tree species on cellulase activity and bacterial community composition in the gut of larval Asian longhorned beetle. Environ. Entomol..

[38] Scully E.D., Geib S.M., Hoover K., Tien M., Tringe S.G., Barry K.W., Glavina del Rio T., Chovatia M., Herr J.R., Carlson J.E. (2013). Metagenomic profiling reveals lignocellulose degrading system in a microbial community associated with a wood-feeding beetle. PLoS ONE.

[39] Scully E.D., Geib S.M., Carlson J.E., Tien M., McKenna D., Hoover K. (2014). Functional genomics and microbiome profiling of the Asian longhorned beetle (*Anoplophora glabripennis*) reveal insights into the digestive physiology and nutritional ecology of wood feeding beetles. BMC Genom..

[40] Ayayee P., Rosa C., Ferry J.G., Felton G., Saunders M., Hoover K. (2014). Gut microbes contribute to nitrogen provisioning in a wood-feeding cerambycid. Environ. Entomol..

[41] Ayayee P.A., Larsen T., Rosa C., Felton G.W., Ferry J.G., Hoover K. (2016). Essential amino acid supplementation by gut microbes of a wood-feeding cerambycid. Environ. Entomol..

[42] Zhang Z., Jiao S., Li X., Li M. (2018). Bacterial and fungal gut communities of *Agrilus mali* at different developmental stages and fed different diets. Sci. Rep..

[43] Gao G., Gao J., Hao C., Dai L., Chen H. (2018). Biodiversity and activity of gut fungal communities across the life history of *Trypophloeus klimeschi* (Coleoptera: Curculionidae: Scolytinae). Int. J. Mol. Sci..

[44] Guo Q., Yao Z., Cai Z., Bai S., Zhang H. (2022). Gut fungal community and its probiotic effect on *Bactrocera dorsalis*. Insect Sci..

[45] Vasanthakumar A., Delalibera I., Handelsman J., Klepzig K.D., Schloss P.D., Raffa K.F. (2006). Characterization of gut-associated bacteria in larvae and adults of the southern pine beetle *Dendroctonus frontalis* Zimmermann. Environ. Entomol..

[46] Delalibera I., Vasanthakumar A., Burwitz B.J., Schloss P.D., Klepzig K.D., Handelsman J., Raffa K.F. (2007). Composition of the bacterial community in the gut of the pine engraver, *Ips pini* (Say) (Coleoptera) colonizing red pine. Symbiosis.

[47] Aylward F.O., Suen G., Biedermann P.H., Adams A.S., Scott J.J., Malfatti S.A., Glavina del Rio T., Tringe S.G., Poulsen M., Raffa K.F. (2014). Convergent bacterial microbiotas in the fungal agricultural systems of insects. Mbio.

[48] Hulcr J., Adams A.S., Raffa K., Hofstetter R.W., Klepzig K.D., Currie C.R. (2011). Presence and diversity of streptomyces in *Dendroctonus* and sympatric bark beetle galleries across North America. Microb. Ecol..

[49] Scully E.D., Hoover K., Carlson J.E., Tien M., Geib S.M. (2013). Midgut transcriptome profiling of *Anoplophora glabripennis*, a lignocellulose degrading cerambycid beetle. BMC Genom..

[50] Mason C.J., Long D.C., Lindroth R.L., Hoover K. (2019). Divergent host plant utilization by adults and offspring is related to intra-plant variation in chemical defences. J. Anim. Ecol..

[51] Mueller R.C., Paula F.S., Mirza B.S., Rodrigues J.L., Nüsslein K., Bohannan B.J. (2014). Links between plant and fungal communities across a deforestation chronosequence in the Amazon rainforest. ISME J..

[52] Caporaso J.G., Lauber C.L., Walters W.A., Berg-Lyons D., Huntley J., Fierer N., Owens S.M., Betley J., Fraser L., Bauer M. (2012). Ultra-high-throughput microbial community analysis on the Illumina *HiSeq* and *MiSeq* platforms. ISME J..

[53] Mogouong J., Constant P., Legendre P., Guertin C. (2021). The *phyllosphere* microbiome of host trees contributes more than leaf phytochemicals to variation in the *Agrilus planipennis* Fairmaire gut microbiome structure. Sci. Rep..

[54] Koski T.M., Zhang B., Mogouong J., Wang H.L., Chen Z.Z., Li H.P., Bushley K.E., Sun J.H. (2024). Distinct metabolites affect the phloem fungal communities in ash trees (*Fraxinus* spp.) native and nonnative to the highly invasive emerald ash borer (AGRILUS PLANIPENNIS). Plant Cell Environ..

[55] Callahan B.J., McMurdie P.J., Rosen M.J., Han A.W., Johnson A.J., Holmes S.P. (2016). DADA2: High-resolution sample inference from Illumina amplicon data. Nat. Methods.

[56] Wang Q., Garrity G.M., Tiedje J.M., Cole J.R. (2007). Naive Bayesian classifier for rapid assignment of rRNA sequences into the new bacterial taxonomy. Appl. Environ. Microbiol..

[57] Schloss P.D., Westcott S.L., Ryabin T., Hall J.R., Hartmann M., Hollister E.B., Lesniewski R.A., Oakley B.B., Parks D.H., Robinson C.J. (2009). Introducing mothur: Open-source, platform-independent, community-supported software for describing and comparing microbial communities. Appl. Environ. Microbiol..

[58] Nguyen N.H., Song Z., Bates S.T., Branco S., Tedersoo L., Menke J.R., Schilling J.S., Kennedy P.G. (2016). FUNGuild: An open annotation tool for parsing fungal community datasets by ecological guild. Fungal Ecol..

[59] Amith M.T., Fujimoto K., Tao C. (2019). NET-EXPO: A gephi plugin towards social network analysis of network exposure for unipartite and bipartite graphs. HCI International 2019-Posters: 21st International Conference, HCII 2019, Orlando, FL, USA, July 26–31, 2019, Proceedings, Part III 21.

[60] Knights D., Kuczynski J., Charlson E.S., Zaneveld J., Mozer M.C., Collman R.G., Bushman F.D., Knight R., Kelley S.T. (2011). Bayesian community-wide culture-independent microbial source tracking. Nat. Methods.

[61] Trivedi P., Leach J.E., Tringe S.G., Sa T., Singh B.K. (2020). Plant-microbiome interactions: From community assembly to plant health. Nat. Rev. Microbiol..

[62] Bin X., Wang P., Shen Y., Xiang X., Jafir M., Wan X. (2024). Investigation of fungal community structure in the gut of the stag beetle *Dorcus hopei* (Coleoptera; Lucanidae): Comparisons among developmental stages. Microb. Ecol..

[63] Veselská T., Švec K., Kostovčík M., Peral-Aranega E., Garcia-Fraile P., Křížková B., Havlíček V., Saati-Santamaría Z., Kolařík M. (2023). Proportions of taxa belonging to the gut core microbiome change throughout the life cycle and season of the bark beetle *Ips typographus*. FEMS Microbiol. Ecol..

[64] Geib S.M., Jimenez-Gasco M.d.M., Carlson J.E., Tien M., Jabbour R., Hoover K. (2009). Microbial community profiling to investigate transmission of bacteria between life stages of the wood-boring beetle, *Anoplophora glabripennis*. Microb. Ecol..

[65] Ziganshina E.E., Mohammed W.S., Shagimardanova E.I., Vankov P.Y., Gogoleva N.E., Ziganshin A.M. (2018). Fungal, bacterial, and archaeal diversity in the digestive tract of several beetle larvae (Coleoptera). Biomed Res. Int..

[66] Grünwald S., Pilhofer M., Höll W. (2010). Microbial associations in gut systems of wood- and bark-inhabiting longhorned beetles (*Coleoptera: Cerambycidae*). Syst. Appl. Microbiol..

[67] Kaltenpoth M., Steiger S. (2014). Unearthing carrion beetles’ microbiome: Characterization of bacterial and fungal hindgut communities across the *Silphidae*. Mol. Ecol..

[68] Scully E.D., Hoover K., Carlson J., Tien M., Geib S.M. (2012). Proteomic analysis of *Fusarium solani* isolated from the Asian longhorned beetle, *Anoplophora glabripennis*. PLoS ONE.

[69] O’Donnell K. (2000). Molecular phylogeny of the *Nectria haematococca-Fusarium solani* species complex. Mycologia.

[70] O’Donnell K., Sutton D.A., Fothergill A., McCarthy D., Rinaldi M.G., Brandt M.E., Zhang N., Geiser D.M. (2008). Molecular phylogenetic diversity, multilocus haplotype nomenclature, and in vitro antifungal resistance within the *Fusarium solani* species complex. J. Clin. Microbiol..

[71] Coleman J.J. (2016). The *Fusarium solani* species complex: Ubiquitous pathogens of agricultural importance. Mol. Plant Pathol..

[72] Aoki T., O’Donnell K., Homma Y., Lattanzi A.R. (2003). Sudden-death syndrome of soybean is caused by two morphologically and phylogenetically distinct species within the *Fusarium solani* species complex—*F*. *virguliforme* in North America and *F*. *tucumaniae* in South America. Mycologia.

[73] Aoki T., O’Donnell K., Scandiani M. (2005). Sudden death syndrome of soybean in South America is caused by four species of *Fusarium*: *Fusarium brasiliense* sp. nov., *F. cuneirostrum* sp. nov., *F. tucumaniae*, and *F. virguliforme*. Mycoscience.

[74] Kolander T.M., Bienapfl J.C., Kurle J.E., Malvick D.K. (2012). Symptomatic and asymptomatic host range of *Fusarium virguliforme*, the causal agent of soybean sudden death syndrome. Plant Dis..

[75] Romberg M.K., Davis R.M. (2007). Host range and phylogeny of *Fusarium solani f.* sp. *eumartii* from potato and tomato in California. Plant Dis..

[76] Jiang Z.R., Masuya H., Kajimura H. (2021). Novel symbiotic association between *Euwallacea* ambrosia beetle and *Fusarium* fungus on fig trees in Japan. Front. Microbiol..

[77] Osborn R.K., Ordóñez M.E., Cognato A.I. (2002). Ecuadorian *Coptoborus* beetles harbor *Fusarium* and *Graphium* fungi previously associated with *Euwallacea* ambrosia beetles. Mycologia.

[78] Aoki T., Kasson M.T., Berger M.C., Freeman S., Geiser D.M., O’Donnell K. (2018). *Fusarium oligoseptatum* sp. nov., a mycosymbiont of the ambrosia beetle *Euwallacea validus* in the Eastern U.S. and typification of *F. ambrosium*. Fungal Syst. Evol..

[79] Brune A., Dietrich C. (2015). The gut microbiota of termites: Digesting the diversity in the light of ecology and evolution. Annu. Rev. Microbiol..

[80] Brune A. (2014). Symbiotic digestion of lignocellulose in termite guts. Nat. Rev. Microbiol..

[81] Wang L., Li C., Wang X., Wang G., Shang S., Dou Z.P., Luo Y. (2022). Gut lignocellulose activity and microbiota in Asian longhorned beetle and their predicted contribution to larval nutrition. Front. Microbiol..

[82] Pham N.Q., Duong T.A., Wingfield B.D., Barnes I., Durán A., Wingfield M.J. (2023). Characterisation of the mating-type loci in species of *Elsinoe* causing scab diseases. Fungal Biol..

[83] Fan X.L., Barreto R.W., Groenewald J.Z., Bezerra J.D., Pereira O.L., Cheewangkoon R., Mostert L., Tian C.M., Crous P.W. (2017). Phylogeny and taxonomy of the scab and spot anthracnose fungus *Elsinoë* (*Myriangiales*, *Dothideomycetes*). Stud. Mycol..

[84] Gañán-Betancur L., Gazis R. (2023). Genome sequence resource of the avocado scab pathogen *Elsinoe* perseae. Microbiol. Resour. Announc..

[85] Baumgartner K., Coetzee M.P., Hoffmeister D. (2011). Secrets of the subterranean pathosystem of *Armillaria*. Mol. Plant Pathol..

[86] Paudyal D.P., Hyun J.W. (2015). Physical changes in satsuma mandarin leaf after infection of *Elsinoë fawcettii* causing citrus scab disease. Plant Pathol. J..

[87] Zhao L., Xiao H., Ma X., Cheng Q. (2020). *Elsinoë australis* causing spot anthracnose on poplar in China. Plant Dis..

[88] Van Heerden A., Pham N.Q., Wingfield B.D., Wingfield M.J., Muro Abad J.I., Durán A., Wilken P.M. (2024). LAMP assay to detect *Elsinoë necatrix*, an important *Eucalyptus* shoot and leaf pathogen. Plant Dis..

[89] Schott J., Rakei J., Remus-Emsermann M., Johnston P., Mbedi S., Sparmann S., Hilker M., Paniagua Voirol L.R. (2024). Microbial associates of the elm leaf beetle: Uncovering the absence of resident bacteria and the influence of fungi on insect performance. Appl. Environ. Microbiol..

[90] Ramírez-Camejo L.A., Zuluaga-Montero A., Lázaro-Escudero M., Hernández-Kendall V., Bayman P. (2012). Phylogeography of the cosmopolitan fungus *Aspergillus* flavus: Is everything everywhere?. Fungal Biol..

[91] Wu S., Wu J., Wang Y., Qu Y., He Y., Wang J., Cheng J., Zhang L., Cheng C. (2022). Discovery of entomopathogenic fungi across geographical regions in southern China on pine sawyer beetle *Monochamus alternatus* and implication for multi-pathogen vectoring potential of this beetle. Front. Plant Sci..

[92] Xiao X., Zhang Y., Wang F. (2021). Hydrostatic pressure is the universal key driver of microbial evolution in the deep ocean and beyond. Environ. Microbiol. Rep..

[93] Delalibera I., Handelsman J., Raffa K.F. (2005). Contrasts in cellulolytic activities of gut microorganisms between the wood borer, *Saperda vestita* (Coleoptera: Cerambycidae), and the bark beetles, *Ips pini* and *Dendroctonus frontalis* (Coleoptera: Curculionidae). Environ. Entomol..

[94] Paludo C.R., Menezes C., Silva-Junior E.A., Vollet-Neto A., Andrade-Dominguez A., Pishchany G., Khadempour L., do Nascimento F.S., Currie C.R., Kolter R. (2018). Stingless bee larvae require fungal steroid to pupate. Sci. Rep..

[95] Schott J., Fuchs B., Böttcher C., Hilker M. (2021). Responses to larval herbivory in the phenylpropanoid pathway of *Ulmus minor* are boosted by prior insect egg deposition. Planta.

[96] Schott J., Jantzen F., Hilker M. (2023). Elm tree defences against a specialist herbivore are moderately primed by an infestation in the previous season. Tree Physiol..

[97] Austel N., Eilers E.J., Meiners T., Hilker M. (2016). Elm leaves ‘warned’ by insect egg deposition reduce survival of hatching larvae by a shift in their quantitative leaf metabolite pattern. Plant Cell Environ..

[98] Cao H., Chen X., Jassbi A.R., Xiao J. (2015). Microbial biotransformation of bioactive flavonoids. Biotechnol. Adv..

[99] Liu R., Bao Z.X., Li G.H., Li C.Q., Wang S.L., Pan X.R., Zhang K.Q., Zhao P.J. (2022). Identification of nematicidal metabolites from *Purpureocillium lavendulum*. Microorganisms.

[100] Corrêa-Moreira D., de Lima Neto R.G., da Costa G.L., de Moraes Borba C., Oliveira M.M.E. (2022). *Purpureocillium lilacinum* an emergent pathogen: Antifungal susceptibility of environmental and clinical strains. Lett. Appl. Microbiol..

[101] Khan M., Tanaka K. (2023). *Purpureocillium lilacinum* for plant growth promotion and biocontrol against root-knot nematodes infecting eggplant. PLoS ONE.

[102] Baron N.C., de Souza Pollo A.Z., Rigobelo E.C. (2020). *Purpureocillium lilacinum* and *Metarhizium marquandii* as plant growth-promoting fungi. PeerJ.

[103] Nguyen N.H., Tamura T., Shimizu K. (2022). Draft genome sequence of *Purpureocillium takamizusanense*, a potential bioinsecticide. Microbiol. Resour. Announc..

[104] Mashilingi S.K., Zhang H., Chen W., Vaissière B.E., Garibaldi L.A., An J. (2021). Temporal trends in pollination deficits and its potential impacts on Chinese agriculture. J. Econ. Entomol..

[105] Lacey L.A., Grzywacz D., Shapiro-Ilan D.I., Frutos R., Brownbridge M., Goettel M.S. (2015). Insect pathogens as biological control agents: Back to the future. J. Invertebr. Pathol..

[106] Panyasiri C., Supothina S., Veeranondha S., Chanthaket R., Boonruangprapa T., Vichai V. (2022). Control efficacy of entomopathogenic fungus *Purpureocillium lilacinum* against Chili Thrips (*Scirtothrips dorsalis*) on Chili Plant. Insects.

[107] Zhao F., Hoffmann A.A., Xing K., Ma C.S. (2017). Life stages of an aphid living under similar thermal conditions differ in thermal performance. J. Insect Physiol..

[108] Berasategui A., Breitenbach N., García-Lozano M., Pons I., Sailer B., Lanz C., Rodríguez V., Hipp K., Ziemert N., Windsor D. (2022). The leaf beetle *Chelymorpha alternans* propagates a plant pathogen in exchange for pupal protection. Curr. Biol..

[109] Ma L.J., Geiser D.M., Proctor R.H., Rooney A.P., O’Donnell K., Trail F., Gardiner D.M., Manners J.M., Kazan K. (2013). *Fusarium* pathogenomics. Annu. Rev. Microbiol..

[110] Teetor-Barsch G.H., Roberts D.W. (1983). Entomogenous *Fusarium* species. Mycopathologia.

[111] Grove J.F., Pople M. (1980). The insecticidal activity of beauvericin and the enniatin complex. Mycopathologia.

[112] Wang Q., Xu L. (2012). Beauvericin, a bioactive compound produced by fungi: A short review. Molecules.

[113] Clevenger K.D., Bok J.W., Ye R., Miley G.P., Verdan M.H., Velk T., Chen C., Yang K., Robey M.T., Gao P. (2017). A scalable platform to identify fungal secondary metabolites and their gene clusters. Nat. Chem. Biol..

[114] Freeman S., Sharon M., Maymon M., Mendel Z., Protasov A., Aoki T., Eskalen A., O’Donnell K. (2013). *Fusarium euwallaceae* sp. nov.—A symbiotic fungus of *Euwallacea* sp., an invasive ambrosia beetle in Israel and California. Mycologia.

[115] Geib S.M., Scully E.D., Jimenez-Gasco M.M., Carlson J.E., Tien M., Hoover K. (2012). Phylogenetic analysis of *Fusarium solani* associated with the Asian longhorned beetle, *Anoplophora glabripennis*. Insects.

