# Supplementary material for: Potential Functions and Transmission Dynamics of Fungi Associated with Anoplophora glabripennis Across Different Life Stages, Between Sexes, and Between Habitats
Source: Insects. 2025 Mar 5;16(3):273. doi: 10.3390/insects16030273 (PMC11943397; doi:10.3390/insects16030273)
Supplement: Supplementary file 1 [file insects-16-00273-s001.zip › Table S3.pdf]

Table S3 Network topology of fungi in the different life stages and associated habitats of *Anoplophora glabripennis*

Samples	Nodes	Edges	Positive correlation ratio (%)	Negative correlation ratio (%)	Ascomycota (%)	Basidiomycota (%)	Mucoromycota (%)
Egg	42	165	98.18	1.92	80.95	16.67	2.38
Female gut	43	77	54.55	45.45	81.4	16.28	2.33
Larval gut	33	38	92.11	7.89	87.88	12.12	0.00
Male gut	49	294	62.24	7.76	79.59	18.37	2.04
Oviposition slit	33	51	80.38	19.62	81.82	18.18	0.00
Frass	29	32	68.75	31.25	89.66	10.34	0.00
